# A CAN-Bus Lightweight Authentication Scheme

**DOI:** 10.3390/s21217069

**Published:** 2021-10-25

**Authors:** Jia-Ning Luo, Chang-Ming Wu, Ming-Hour Yang

**Affiliations:** 1 Department of Computer Science and Information Engineering, Chung Cheng Institute of Technology, National Defense University, Daxi District, Taoyuan City 335009, Taiwan; 2 Department of Electronic Engineering, Chuan Yuan Christian University, Chung-Li District, Taoyuan City 320314, Taiwan; cmwu@cycu.edu.tw; 3 Department of Information and Computer Engineering, Chuan Yuan Christian University, Chung-Li District, Taoyuan City 320314, Taiwan

**Keywords:** CAN bus, message authentication code, one-time password

## Abstract

The design of the Controller Area Network (CAN bus) did not account for security issues and, consequently, attacks often use external mobile communication interfaces to conduct eavesdropping, replay, spoofing, and denial-of-service attacks on a CAN bus, posing a risk to driving safety. Numerous studies have proposed CAN bus safety improvement techniques that emphasize modifying the original CAN bus method of transmitting frames. These changes place additional computational burdens on electronic control units cause the CAN bus to lose the delay guarantee feature. Consequently, we proposed a method that solves these compatibility and security issues. Simple and efficient frame authentication algorithms were used to prevent spoofing and replay attacks. This method is compatible with both CAN bus and CAN-FD protocols and has a lower operand when compared with other methods.

## 1. Introduction

**Figure 1:**
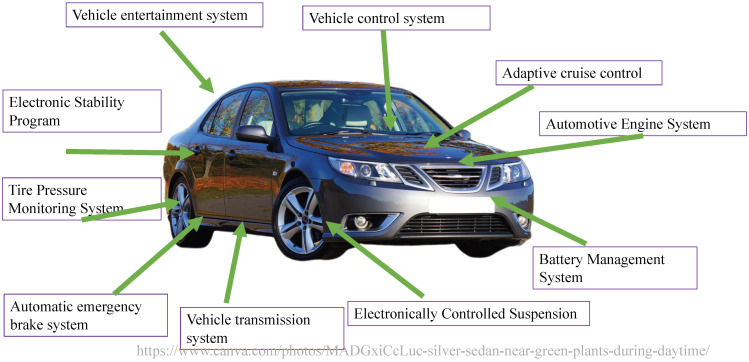
Automotive architecture systems of modern cars.

Automotive architecture systems have become increasingly complex and diverse. In response to people’s demands related to automotive safety, comfort, and entertainment systems, numerous electronic components have been added to vehicles. Conventional end-to-end communications are no longer adequate for the effective management and use of these devices, and in-vehicle networks have gradually become mainstream. An in-vehicle network comprises vehicles outfitted with multiple interconnected processors, coordinating tasks, and shared messages. An automotive bus is the communication network that interconnects underlying automotive devices or automotive instruments in the in-vehicle network [1]. Currently, four mainstream automotive buses are used: Controller Area Network (CAN bus) [2], Local Interconnect Network (LIN) [3], FlexRay [4], and Media Oriented Systems Transport (MOST) [5]. These four automotive buses have different transfer speeds and characteristics on the basis of various applications and requirements. Modern cars are typically equipped as follows, shown in Figure 1.

Among these protocols, MOST was developed for in-vehicle use and services multimedia applications. MOST transfer speeds rates are greater than 10 Mbps, which can meet the demands of various communication and entertainment devices but is costly. Currently, it is only used by multimedia devices to transmit information. FlexRay is a set of communication standards jointly developed by a consortium comprising BMW, Philips, Freescale, and Bosch. FlexRay uses time- and event-triggered communications in which all nodes must be synchronized. The advantages of FlexRay are its speed and reliability; therefore, FlexRay is mostly used in X-by-wire systems with high real-time requirements. LIN is based on universal asynchronous receiver-transmitters and serial communication interfaces and is a low-cost serial communications network that uses single master and multiple slave models. Defined for automotive distributed electronic systems, LIN is mostly used for opening and closing doors and windows and in lighting equipment.

CAN bus was originally designed by Bosch in 1986 for automotive monitoring and controls and was mainly used in communications between the measurement and execution components of a car [6]. Transfer speeds can be as high as 1 Mbps (for communication distances shorter than 40 m) with a payload limited to eight bytes; in vehicular use, transfer speed is typically 500 kbps and can be as low as 5 kbps. The CAN bus transfer medium can be twisted pair or coaxial cabling, which are cost-effective and resistant to electrical interference and can perform self-diagnostics and error corrections. CAN bus can be used in in-vehicle network real-time systems, mostly in essential engine and braking system components, and are currently mainstream in in-vehicle networks [1]. CAN buses do not distinguish between master and slave nodes, and any node can initiate data communications with any other node; the communication order is determined by the priority of each node’s message. When multiple nodes initiate sessions simultaneously, low-priority messages must yield to high-priority messages to prevent congestion of the communication lines.

In terms of transfer speeds, CAN bus can be further distinguished into high-speed or low-speed CAN (CAN-C or CAN-B, respectively) [7]. The standard for CAN-C is ISO11898-2 [8]; the transfer speeds are 125 kbps to 1 Mbps and, because it can satisfy the real-time data transfer requirements of drive systems, CAN-C is often used to connect and control engine, transmission, and dashboard systems. The CAN-B standard is ISO11898-3 [6]; CAN-B has transfer speeds of 40–125 kbps and is mostly used for leisure and chassis control systems.

In a CAN bus, each node is an electronic control unit (ECU). CAN bus involves communication between ECUs through broadcasts. For every CAN bus frame, the circumstance for not containing bit stuffing is 111 bits, namely a 1-bit start of frame (SOF), which denotes the start; an 11-bit identifier (ID) field (the expanded format is 29 bits); a 10-bit remote transmission request, which is used to indicate whether to accept the data; a 1-bit identifier extension bit, which is used to indicate that the data frame is either a base or an extended format; a 4-bit data length code (DLC), which indicates the length of the data; a 64-bit data field; a 16-bit cyclic redundancy check (CRC) field, with the last 1 bit as the CRC delimiter; a 2-bit acknowledge slot (ACK), with the later 1 bit as the ACK delimiter and expressed as 1 for transmitter or 0 for receiver; and a 7-bit end-of-frame (EOF) for concluding the frame.

**Figure 2:**
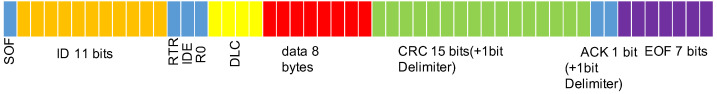
CAN bus frame format.

In each frame, the identifier field is the identification code. When multiple ECUs are about to transmit information simultaneously, the CAN bus arbitration mechanism uses the ID to determine the priority of the data transmissions—when multiple frames are being sent out simultaneously, the frame with the smallest ID are sent out first, and no host is required to control the destination of the transmission frames, facilitating their rapid transmission. The CAN bus frame format is presented in Figure 2.

**Figure 3:**
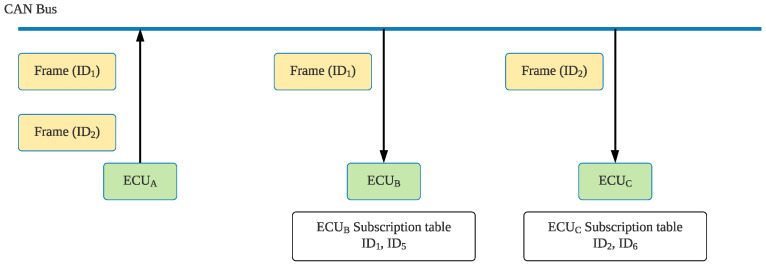
CAN bus process for transmitting frames.

The CAN bus process for transmitting frames is presented in Figure 3. Because CAN does not involve addresses for nodes in the network and because the frame ID only denotes the frame type, the CAN nodes are unaware of the CAN configuration. Therefore, each ECU contains a receiving list that stores the frame IDs that must be received. When $ECU_{A}$ transmits a frame to the CAN bus, all other ECUs receive the frame signal. Each ECU then checks whether the frame ID is a signal that they must receive; if so, they will receive the frame. Figure 3 assumes that $ECU_{B}$ must receive $ID_{1}$ and $ID_{5}$ frames; therefore, these two IDs appear on the $ECU_{B}$ receiving list. When $ECU_{A}$ sends out a frame identified as $ID_{1}$ to the CAN bus, $ECU_{B}$ checks its receiving list, and after it determines that $ID_{1}$ is a frame that it requires, it receives the information for the frame. This type of frame model facilitates ECU deployment flexibility, and if the ECU nodes require new frame types, the corresponding frame IDs can be directly added to their receiving list. When new nodes are added, they can be directly connected to the network without modifying other nodes in the network.

CAN buses are designed with an automatic error detection mechanism that sends out different error frames on the basis of five errors. The first type of error is bit error, wherein the transmitter compares the transmitted data with the data presented to the bus to check for inconsistencies; if consistencies are detected, an error frame is sent out directly after the message is transmitted. This error detection mechanism can only detect SOF, DLC, data, and CRC errors. The second type of error is stuff error, wherein on the CAN bus, transmitters and receivers agree that, after several consecutive identical bits, an opposite bit is sent to differentiate them from certain delimiters in the message. CAN data can have at most five identical bits, and an opposite bit is automatically inserted as the next bit. CAN only has a bit stuffing mechanism for unfixed fields such as SOF, ID, DLC, data, and CRC; when six identical bits are consecutively detected in these fields, an error frame is sent out starting from the next bit. The third type of error is CRC error, wherein CRC is a checksum mechanism used to detect errors that may arise after data transmission. The generated numbers are calculated before transmission and attached to the end of the data. Later, the receiver tests and confirms whether any changes to the information occurred. If the receiver detects CRC errors in the frame, it sends out an error frame. The fourth type of error is form error, wherein in addition to the CRC and ACK delimiters, EOF is the delimiter for the entire frame. The preset value of these three fields is 1. If these bits are 0, an error frame is sent out starting from the next bit. The fifth type of error is acknowledgment error, wherein the transmitter sends out a 1 for the ACK slot and 1 for the ACK delimiter, and the receiver will return 0 s; if no response is received, an error frame is sent out after the ACK delimiter. Every ECU in the CAN bus has a counter that records the number of errors; when an error appears in the ECU, the number increases, and when no errors appear in the ECU, the number decreases. When the count exceeds 255, the ECU is disabled, and all the node’s behaviors are stopped until the node detects specified data to reinitialize the node, reset the counter, and return them to the bus. Table 1 presents the types of error frames.

CAN bus is a closed network, and even if the network protocol itself has numerous security loopholes, driving safety should not be affected. However, Hoppe and Dittman reported a CAN bus cyber-attack on electric windows in 2007 [9]. Since then, numerous scholars have published papers on CAN bus cyber-attacks, which are mainly distinguished into two types. These cyber-attacks targeting the sensing and communication layers can compromise the security of the control layer of the car [10]. The first type is a physical access attack, which involves the attacker using physical circuits to connect to networks in the vehicle; however, because this type of attack requires physical contact with the vehicle, it was not initially considered. Later, in response to consumer demands for in-vehicle information and entertainment systems, car vendors began producing customized entertainment and control environments, and some internal car systems became accessible through Bluetooth or WiFi. For example, BMW provided an iPod-out connector in 2011, enabling the driver to play music from their iPod or iPhone. Furthermore, most modern car models all have USB, WiFi [11], or Bluetooth [12] connections for phones or other mobile devices. These devices mostly connect with the CAN bus and can control different systems. Attacks can exploit loopholes in these systems or communication protocols, enabling individuals to access the vehicle remotely and take complete control of it [13]. This poses a serious risk to vehicle safety. These types of attacks carried out through wireless communications are called remote access attacks, which is the second type of attack. Most relevant studies have focused on remote access attacks.

Currently, remote access attacks are mainly distinguished into denial-of-service attacks and message tampering.

1. Denial-of-service attacks: An attacker can use the CAN bus arbitration mechanism to inject numerous highest priority message frame IDs, inhibiting the transmission of messages from other ECUs. The attacker can also use error frames to force certain ECUs to become bus-off [14], paralyzing their service. For specific systems (e.g., tire-pressure monitoring systems), their sensors can be connected into an ad-hoc network to initiate wormholes or blackholes to suspend their services [15,16]. In addition, attacks can install malware to paralyze services [17].
2. Message tampering: This involves taking control of ECUs by using disguised IDs or fake frames or by conducting replay attacks by replaying a prerecorded frame. Replay attacks cause the receiver to receive an incorrect frame. Because of the plaintext transmissions of park assist systems, the speed and angle of entry can be easily parsed, and consequently, attackers can cause collisions during assisted parking by sending out frames with erroneous speed or angles or by replaying erroneous frames [11]. Our main focus is on message tampering.

Remote access attacks can be distinguished into two types of ports. The first involves accessing the central gateway through the vehicle’s on-board diagnostics II (OBD-II) to access the central gateway. The second involves accessing the CAN bus through multimedia entertainment devices, which leads to apparent CAN bus security risks. Attacks through OBD-II and the entertainment system are described subsequently.

1. OBD-II was originally intended to enable vehicle maintenance personnel to diagnose the vehicle’s state. Through the OBD interface, maintenance personnel can use the CAN bus to read the status information of every ECU in the vehicle and diagnose any malfunctioning parts. Before the prevalence of OBD-II and the existence of Bluetooth and smart phones, drivers could not conveniently connect devices to the OBD-II. Manufacturers have since added Bluetooth pairing, enabling drivers to see the status of the car’s CAN on their phone. However, this Bluetooth portal has led to an increase in attacks. The OBD interface was not designed with access control mechanisms, and consequently, anyone can obtain information about the vehicle through the OBD-II and even control the ECUs of the attacked vehicle [11,18,19,20]. For example, Miller et al. [11] injected false frames into a CAN bus by using the OBD interface, enabling them to shut down the engine, activate the accelerator, and even control the vehicle’s speed and direction while the vehicle was moving.
2. Entertainment systems: In-vehicle entertainment systems are combinations of hardware and software that provide music or video entertainment in a vehicle. In-vehicle entertainment began with sound systems consisting of a radio and a cassette or CD player; these systems now include navigation systems and video players as well as USB, Bluetooth, and WiFi connectivity. In addition to installing malware on CDs, a smart phone, or other removable devices, attacks can install malicious code by using Bluetooth or telephone networks, and even a CAN signal can be used to gain complete control of an ECU [13]. Victims typically do not notice attacks being conducted through Bluetooth or telephone networks, and these types of attacks can spread quickly and be conducted over the internet, producing considerably substantial effects, impacts, and threats. Table 2 presents the routing methods of CAN bus data packets and ECU updating methods used to gain complete control of a vehicle.

In this study, a CAN bus-based security improvement mechanism is proposed to prevent attacks through message tampering, which involves attackers sending erroneous frames through an OBD-II or entertainment system. Additional MACs can be used for authentication, and replay attacks can be prevented by adding a time counter when creating frame authentication codes. Section 2 introduces CAN bus authentication methods proposed by other scholars. Section 3 describes the methods and principles used in this study, and Section 4 explains the overall system and protocols. Section 5 presents the security analysis of the attack preventions, which is followed by the conclusion.

## 2. Related Works

Numerous methods for preventing CAN bus message tampering have been proposed, such as *CAN Frame Authentication* and *CAN Frame Encryption* [21]. Because data has only 8 bytes, some methods send data in several packets. There are also some methods to limit the size of the original data, and then use the remaining space to add authentication information.

In 2008, Nissen et al. proposed an delayed-authentication mechanism by using 64-bit message authentication code [22]. The 64-bit MAC of the first four messages is sent in the subsequent four messages. Therefore, if the attacker fakes the message, the system will not find out until the next four packets are received.

In 2012, Hartkopp et al. proposed MaCAN [23]. They argued that CAN bus is vulnerable because its frames do not have source or target addresses. Therefore, the researchers recommended designing another 6-bit ID for each ECU as the address sending out both the source and target IDs. They proposed sending out a signed message every few frames to verify that the preceding frames are trustworthy and employing time synchronization to prevent replay attacks. To periodically synchronize the time, the authors added a time server to the system; at regular intervals, the ECUs send a request to the time server, and after receiving the request, the time server sends a timecode and the authenticated MACs to the ECU. However, the frame itself is not used for authentication; rather, the signature sent every few frames would be used. If high-frequency authentication is required (e.g., sending a signature with each frame), the transmission volume of the CAN bus doubles, and the CAN bus loses its real-time feature. Furthermore, CAN bus time units are not particularly precise and can easily become desynchronized. Because of the inherent nature of CAN, only one ECU can request a timecode at a given time; when the ECU receives a response, it begins keeping time again, but differences in time flow may cause desynchronization.

In 2014, Woo et al. [24] proposed sectioning MACs into the CRC field and the position of the expanded ID, 32 bits in total, and adding a counter to each ECU to prevent replay attacks. When recreating the group key, the counter is reset to zero. These described methods mostly use counters to prevent replay attacks, but counters may become desynchronized because of the loss of packets, requiring a substantial amount of resources to synchronize them in the CAN bus. In the CaCAN [25] system, the data field in the original packet will be reduced. Then the authentication message will be added to the subsequent packet.

In 2015, Ueda et al. [26] which uses a MAC on the basic framework and attaches it to the second frame, which is sent out immediately after the original frame. The receiver must receive both the original frame and the MAC frame to be authenticated. In 2016, Nürnberger et al. proposed vatiCAN [27], which uses the same concept of [26]. In vatiCAN, the receiver must receive both the original frame and the MAC frame to be authenticated. To prevent replay attacks, a time-incrementing counter is added to each ECU. A *nonce generator* (NG) is then added to periodically broadcast a nonce; ECUs subsequently set their counters to the new value they receive and become synchronized. To prevent the frames from colliding during the arbitration phase, the IDs of the authenticated frames are set to the original frame ID plus 1, which is intended to help the authenticated frame avoid arbitrary collisions from other frames. Consequently, the authentication ID and the original frame ID are very similar; when the original frame passes authentication, it can also pass arbitration. However, nonces generated with an NG may be smaller than those of the original counter, and at this time, the ECU is susceptible to replay attacks and must wait for a second authenticated frame, losing its convenient, real-time properties.

Later designs integrated the authenticated information and the original frame in the same frame. For example, mini-CAN [28] (2017) claims that 60% of frames in in-vehicle networks only use 4 bytes of information fields and that MACs can be placed in the remaining, unused 4 bytes. Other methods use frames exceeding 4 bytes, which in their assessment do not require authentication. They proposed a MAC built from historical frames, specifically the original frame, the counter, and the 16 frames recorded in advance. After a key was used to create the hash-based MAC (HMAC), it was cut down to 4 bytes to form a MAC. This method cannot be replayed and therefore can be used to prevent spoofing attacks. In the current study, keys were distributed at the beginning, and no exchanges were allowed afterwards. An ECU shares a key with any ECU that engages in a communication session, and this key is saved in the memory of both ECUs in advance. Because of the difficulty involved in ensuring that every frame is successfully accepted by the CAN bus, if any party fails to receive a frame, the 16 frames that were recorded in advance cannot be synchronized, and they will be unable to authenticate the MAC.

In consideration of all frames (i.e., instead of using only frames that use 4 bytes), PreAuthCode [29] (2020) was proposed, which places the MAC in the position of the expanded ID. Each time a frame is sent, the session key and the current counter are used to create the HMAC, which is sent out using the truncated initial 18 bits and information. This can help prevent replay and spoofing attacks. All transmitters and receivers share a symmetric key. During initialization, the transmitter generates a 32-bit seed, which is used with the preshared key to create an HMAC; the first 32 bits are then transmitted. After the receiver has successfully received and authenticated the MAC, both parties use the seed and 0 to create the first session key. The transmitter uses the session key and its own ID to create an HMAC and directly send 64 bits back to the transmitter to demonstrate that the seed was received. The session key is updated when the counters of both ends have reached the specified threshold; both parties then use a key and the number of current sessions to create the next session key. However, if any ECU counter is desynchronized, the other counter will no longer be synchronized. Consequently, the timing for updating session keys will not be synchronized, and the ECUs will no longer be able to authenticate MACs.

In 2016 Radu proposed an AUTOSAR [30,31,32] compliant lightweight authentication protocol for CAN [33]. CANAuth [34] and LiBrA-CAN [35] are two protocols for light-weight authentication over CAN+ [36].

Other methods use fingerprint, fuzzy system or machine learning to detect the attack [18,37,38,39,40,41,42]. For example, in 2016, Cho et al. proposed a Clock-based IDS (CIDS), which uses the fingerprinting method to measure and exploit the intervals of periodic in-vehicle messages [18]. However, CIDS cannot detect the original of the attack message if the attacker injects messages periodically. In 2020, Yang proposed a method that use a RNN with the LSTM unit to extract the deep features of the analog CAN signal to detect the spoofing attack on the CAN bus [40]. Zhang et al. also proposed the *CANsec* [41], a security evaluation tool that simulates malicious attacks to evaluate the security risk of the in-vehicle CAN. In 2021, Andersson proposed an intrusion detection method that combines anomaly and signature based algorithms [42]. However, in MaCAN [23], a highly precise time is required, which is extremely reliant on time synchronization. Components in CAN are also generally very inexpensive, and timing precision is a concern.

Therefore, we proposed and designed an authentication method that effectively prevents replay attacks and does not require a massive amount of resources for synchronization by using the message authentication code (MAC). Due to the limitation of the data field of a CAN frame, which is limited to 8 bytes, we use the 15-bit CRC field to transmit the MAC value.

Moreover, the future development of the CAN are CAN-flexible data rate (CAN-FD) [43]. In CAN-FD, the transmission speed can be up to 8 Mbit/s, and the amount of data in a CAN-FD frame is increased from 8 to 64 bytes. Moreover, the CAN-XL protocol [44] increases the payload up to 2048 bytes and the transmission rate up to 10 Mbit/s. Our method can also be applied in CAN-FD and CAN-XL environments, even in systems that run CAN bus and CAN-FD in parallel. The proposed method is anticipated to be successfully implemented on CAN-FD.

## 3. Method and Design

To improve the security of the CAN bus and to maintain compatibility with CAN bus protocols, we replaced the CRC field with our proposed MAC but did not alter the data field sent to the CAN. We then used a general MAC with a minimum required data length of 64 bits (when using MD-5 algorithm [45]) and a maximum length of 512 bits (when using SHA2-512 [46]). However, the CRC field on the CAN only has 15 bits; therefore, we proposed the new message authentication mechanism described subsequently.

### 3.1. Message Authentication Code (MAC)

**Figure 4:**
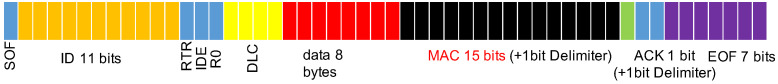
CAN bus frame with the MAC field.

We added an message authentication code in each frame to prevent frame spoofing and then used HMAC-Based One-Time Password (HOTP), a mechanism released on RFC4226 by the Internet Engineering Task Force in 2005 [47], to reduce the calculated MAC to the appropriate size for placement into the 15-bit CRC field, as shown in Figure 4).

#### 3.1.1. Counter-Based MAC

We first assumed that the transmitter and the receiver both have the key *K* and a counter that increases when a message is received. We also assumed that the transmitter is $C_{A}$, the receiver is $C_{B}$, and the CAN frame is *M*, which contains the fields ID, DLC, and data. The transmitter uses one-way hash function SHA1-160 [48] to calculate the MAC. SHA1-160 results are 160 bits; therefore, we expressed the results as an H[0:19] byte array. However, the CAN CRC field only has 15 bits; therefore, we used the lowest 4 bits of the last byte of H[0:19] as the *offset* (which is in the range of 0 to 15). We then took two bytes from the byte array H[0:19], which were H[offset:offset+1]:

$$\begin{array}{c} H\left[0:19\right]={\mathrm{SHA}}_{160}(K∥C_{A}∥M)\\ \mathrm{offset}=H[19]\&0x0f. \end{array}$$

Because of the characteristics of one-way hash functions, the offset value after each calculation is not fixed. Therefore, our method ensures that for each calculation, the extracted data is a random value. When extracting H[offset:offset+1], the most significant bit (MSB) is ignored to obtain the MAC value:

$$\mathrm{MAC}=\left(H\left[\mathrm{offset}\right]*256+H\left[\mathrm{offset}+1\right]\right)\;\mathrm{mod}\;2^{15}.$$

**Figure 5:**
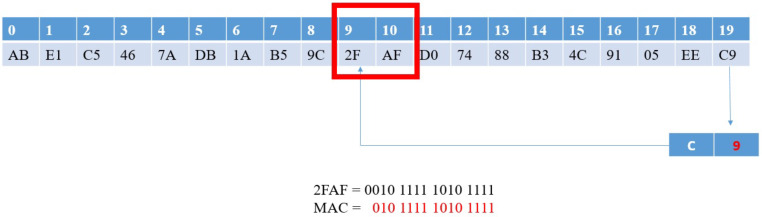
MAC generation.

Figure 5 presents the MAC generation schematic.

Next, the calculated MAC can be added to the original CAN bus’s CRC field. Before the message is sent out, the timer is increased by 1. When the receiver receives the frame, it performs the following calculation: Use one-way hash function HMAC to calculate the MAC value:

$$H^{\prime }={\mathrm{SHA}}_{160}\left(K|\right|C_{B}||M).$$

When taking H’[offset: offset + 1], ignore the MSB to obtain the following:

$$\mathrm{MAC}’=\left(H^{\prime }\left[\mathrm{offset}\right]*256+H^{\prime }\left[\mathrm{offset}+1\right]\right)\;\mathrm{mod}\;2^{15}.$$

Next, extract the MAC in the message. If the calculated MAC’ and the extracted MAC are the same, the message passes verification. After that, the receiver increases its counter *K* by 1.

However, in the CAN bus, the transmitter’s and receiver’s counters cannot be synchronized, and in the event of a missing packet, both parties become desynchronized. Therefore, we used system time to replace the counter to calculate the MAC.

#### 3.1.2. Time-Based MAC

We assume that each ECU in the vehicle has microcontrollers with time units. Although the CAN network does not contain any network time protocols, because current microcontrollers are designed with timers, the timer values return to zero when the system is reset and increase with each subsequent second. This incremental timer is the time we used.

This time, the transmitter and the receiver both hold the key *K*. The current time for the transmitter is $T_{A}$, the current time for the receiver is $T_{B}$, and the message is *M*. The transmitter uses one-way hash function HMAC to calculate the MAC:

$$H\left[0:19\right]={\mathrm{SHA}}_{160}(K∥T_{A}∥M).$$

Next, the offset is calculated, which yields:

offset=H[19]&0x0f.

Next, the MAC is calculated:

$$\mathrm{MAC}=H\left[\mathrm{offset}:\mathrm{offset}+1\right]\;\mathrm{mod}\;2^{15}.$$

Then, the MAC is added to the CRC field of the original CAN frame and is sent out as a packet.

When the receiver receives the frame, *M* is composed from the frame ID, DLC, and data; the following verification is performed using the receiver’s own current time $T_{B}$:

$$H’={\mathrm{SHA}}_{160}(K∥T_{B}∥M).$$

When extracting H[offset: offset + 1], the MSB is ignored to obtain:

$$\mathrm{MAC}’=\left(H\left[\mathrm{offset}\right]*256+H\left[\mathrm{offset}+1\right]\right)\;\mathrm{mod}\;2^{15}.$$

Finally, the message MAC is extracted. If the calculated MAC’ is the same as the extracted MAC, the message passes verification. The advantage of employing time synchronization is that neither resources nor time are not required for synchronization. However, the disadvantage is that messages in the same second can be recorded and replayed, and the transmitter time $T_{A}$ and receiver time $T_{B}$ may be different. Therefore, we improved the time synchronization method.

#### 3.1.3. Time Intervals

To reduce the possibility of replay attacks and to solve the problem of transmitter time $T_{A}$ and receiver time $T_{B}$ being different, we used time counter TC=(T)/Δt, with Δt as the custom time interval. This value can be adjusted on the basis of the use requirements and safety. Even when $T_{A}$ and $T_{B}$ are different, the time counter can be synchronized, enabling the successful authentication of the message; Δt can also be reduced to reduce the possibility of replay attacks. Constantly calculating the time counter within the time interval is also unnecessary, and omitting this can effectively reduce resource waste.

This time, the transmitter and the receiver both hold the key *K*. The current time for the transmitter is $T_{A}$, the current time for the receiver is $T_{B}$, and the message is *M*. First, calculate the time counter $TC_{A}$:

$${\mathrm{TC}}_{A}=T_{A}/\Delta t.$$

Use one-way hash function HMAC to calculate the MAC:

$$H={\mathrm{SHA}}_{160}(K∥{\mathrm{TC}}_{A}∥M).$$

Next, cutting with the aforementioned method yields the following:

$$\begin{array}{c} \mathrm{offset}=H[19]\&0x0f\\ \mathrm{MAC}=\left(H\left[\mathrm{offset}\right]*256+H\left[\mathrm{offset}+1\right]\right)\;\mathrm{mod}\;2^{15}. \end{array}$$

Next, the MAC is added to the original CAN frame’s CRC field. When the receiver receives the frame, it performs the following authentication: First, the time counter $TC_{B}$ and the message digest are calculated:

$$\begin{array}{c} {\mathrm{TC}}_{B}=T_{B}/\Delta t\\ H’={\mathrm{SHA}}_{160}(K∥{\mathrm{TC}}_{B}∥M). \end{array}$$

When extracting H’[offset: offset + 1] , the MSB is eliminated, yielding:

$$\mathrm{MAC}’=\left(H’\left[\mathrm{offset}\right]*256+H’\left[\mathrm{offset}+1\right]\right)\;\mathrm{mod}\;2^{15}.$$

Finally, the message MAC is extracted. If the calculated MAC’ is the same as the extracted MAC, the message passes verification. However, this mechanism has two remaining problems:

1. In situations when the frame’s own transmission time—that is, $T_{B}-T_{A}>\Delta t$, which exceeds a time interval, causing the time difference between the transmitter and the receiver to exceed a time interval, the time counters become desynchronized and fail authentication;
2. Each ECU in the CAN may have errors in their time unit. When the vehicle is set, the time error of each ECU gradually increases until finally the time counters become desynchronized and fail authentication. Solutions to these two problems are described subsequently.

#### 3.1.4. Time Counter Synchronization and Offset

In consideration of possible time discrepancies when ECUs receive transmissions from the CAN bus, which leads to desynchronized time counters, if the calculated MAC is not the same, the receiving node $ECU_{B}$ corrects the time through the following method:

$${\mathrm{TC}}_{B}=T_{B}/\Delta t+\mathrm{error}+\mathrm{drift}.$$

**Figure 6:**
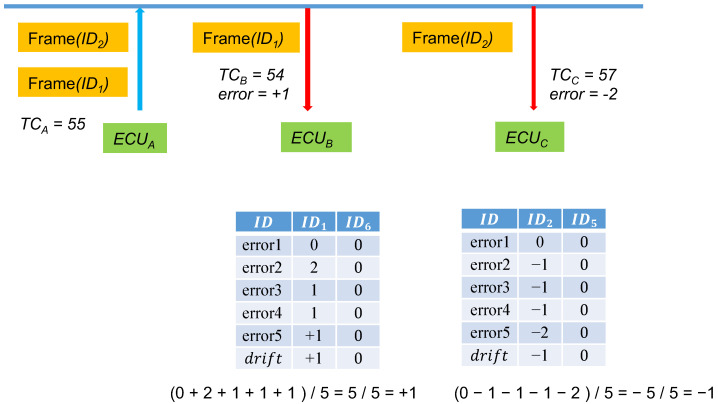
Logging *drift* into the storage tables.

$ECU_{B}$ compares the MACs using the aforementioned method in the following order: ${\mathrm{TC}}_{B}+1,{\mathrm{TC}}_{B}-1,{\mathrm{TC}}_{B}+2,{\mathrm{TC}}_{B}-2$, and so on until reaching ${\mathrm{TC}}_{B}+n$ and ${\mathrm{TC}}_{B}-n$, where *n* is the set threshold that can be adjusted on the basis of usage requirements and security. We named this job error. Furthermore, a time offset value, drift, was set with an initial value of 0. After several errors are collected, drift is set to the average of the errors as the baseline for the subsequent message synchronization. Figure 6 explains the drift storage. When its own time counter is 55, $ECU_{A}$ sends out a frame, and when $ECU_{B}$ receives the frame, its own time counter is 56. At this time, $TC_{B}$ = 56 will not result in a successful authentication, and therefore, $ECU_{B}$ will begin testing by using ±n to calculate the following:

$$\begin{array}{c} H_{1}={\mathrm{SHA}}_{160}(K∥{\mathrm{TC}}_{B}∥M)\\ H_{2}={\mathrm{SHA}}_{160}(K∥{\mathrm{TC}}_{B}+1∥M)\\ H_{3}={\mathrm{SHA}}_{160}(K∥{\mathrm{TC}}_{B}-1∥M)\\ H_{4}={\mathrm{SHA}}_{160}(K∥{\mathrm{TC}}_{B}+2∥M)\\ H_{5}={\mathrm{SHA}}_{160}(K∥{\mathrm{TC}}_{B}-2∥M). \end{array}$$

Until all the values between ${\mathrm{TC}}_{B}+n$ and ${\mathrm{TC}}_{B}-n$ have been tested.

In the example, after ${\mathrm{TC}}_{B}-1$ has been tested, because it is equal to 55, the authentication is successful. Consequently, the calculated error is −1.

Here, five instances are the frequency for calculating averages. The yellow cells record the errors of the previous four instances and the fifth instance. At this time, $ECU_{B}$ calculates the average of the errors of the five message transmissions and then saves −1 in the drift field of $ID_{1}$. The next time the $ID_{1}$ frame is received, $ECU_{B}$ finds $ID_{1}$ from the table and determines that the drift value is −1; calculations based on the current ${\mathrm{TC}}_{B}-1$ are directly performed. After the first correction, a massive amount of time can be saved in calculating authentication codes.

In the proposed system, the Δt value was set to 1 s. Therefore, in the poorest comparison scenario (i.e., the error value is ±n), the range of the time error between the two ECUs is 2n + 1 seconds.

When Δt is high, because the allowable time error value increases, packets within the range of Δt+drift+error may be subject to replay attacks. Errors may also be present in the ECU timing unit (e.g., it may be one second off). Consequently, we adjusted the drift.

**Figure 7:**
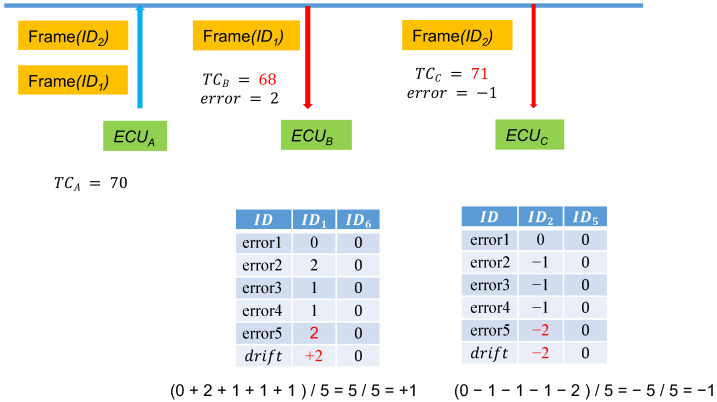
Updating the *drift* in the storage tables.

During the first few messages, $ECU_{B}$ calculates the drift between the two nodes to synchronize the time. In later messages, $ECU_{B}$ only accepts the drift ± error value to minimize the threat of replay attacks. If the drift corrections of the messages for a subsequent period of time are all identical, as depicted in Figure 7, when $ECU_{B}$ observes that drift’ = 1 according to the past several message errors for $ID_{1}$ messages, then the drift for $ID_{1}$ is reset to the original drift + 1 = 2. By continuously correcting the drift value, even as the time error between $ECU_{A}$ and $ECU_{B}$ increases, sessions can be held in situations of reduced replay attacks.

Furthermore, because MACs are only 15 bits, attacks only require 215 attempts to guess a possible MAC for a specific message. However, because attackers do not know the current time counter and the shared key K, they cannot deduce the MAC of the next address. Even if the attackers are monitoring the CAN network, they cannot deduce the shared key K and the corresponding MAC.

### 3.2. Key Distribution

Gateways were added to the proposed system to distribute the same group key to ECUs within the same group. During a session, this key generates a MAC for authentication. The process of creating a group key involves the following steps:

(A) System initialization. Before the vehicle leaves the factory, the gateway is set to share a key with individual ECUs in the subnet. This key is only used when adding or removing devices. The gateway shares the same group key with all the ECUs in the subnet, and this group key is used to generate session keys.
(B) System reset. When the system is reset, the gateway resets a frame to the ECU group. After receiving this frame, each ECU resets their time counter to zero, and the time counter begins increasing after each time interval. This frame is not attached to a MAC because all the ECUs only receive this frame at the system reset, and not afterwards. Unless the system is reset, even if an attacker sends a reset frame again, the remaining ECUs ignore it.
(C) Initial distribution of keys. After the gateway resets the time counters to zero, it generates a seed used to create group keys. The gateway then uses this seed to create a MAC for the group key shared with every ECU in advance and then broadcasts the MAC to each ECU.
(D) Key updates. Occasionally, the gateway generates the seed for the next group key and then uses the previous group key to create a MAC. This frame is sent to each ECU in the form of a broadcast.
(E) Adding an ECU device. When a new ECU is added, the group key is not resent. However, the new device does not have the previous group key; therefore, the gateway first sends a new encrypted group key to the new device and then sends an encrypted time counter. Subsequently, the gateway updates the key for the remaining ECUs with a new seed. Therefore, the new device can send frames to the other ECUs. The group key that was stored in advance is then changed to the current group key, enabling the new device to update its key.
(F) Removing an ECU device. When an ECU is being removed, because the ECU has the previous group keys, the gateway was allowed to use the key that was shared with every ECU in advance to send a new encrypted group key. The removed ECU cannot decrypt the new group key, even with the old group key. At this time, the prestored group key is changed to the current group key, and the removed device is therefore unable to update its key.

## 4. System and Protocol

### 4.1. System Framework: CAN Bus Partitions

**Figure 8:**
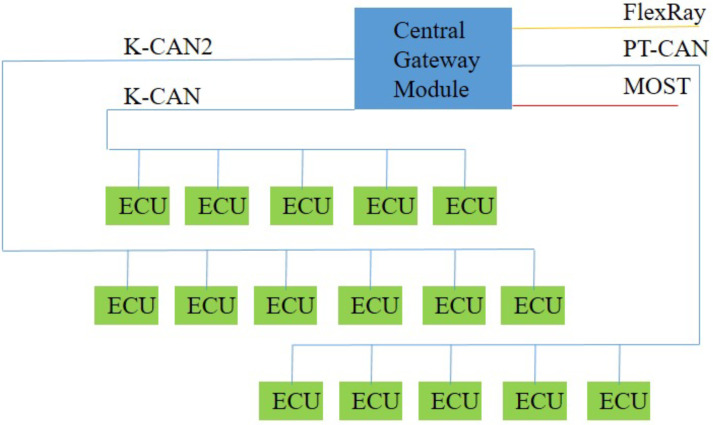
Framework of an in-vehicle network.

In actual in-vehicle networks, the CAN bus network does not connect all the ECUs together, but rather divides them into several high-speed and low-speed CAN bus networks connected by a central gateway module. Other heterogeneous networks, such as FlexRay and MOST, are also connected through gateway modules, as depicted in Figure 8. High-speed CAN bus networks connect the vehicle’s core control system, whereas low-speed CAN bus networks connect the peripheral systems.

**Figure 9:**
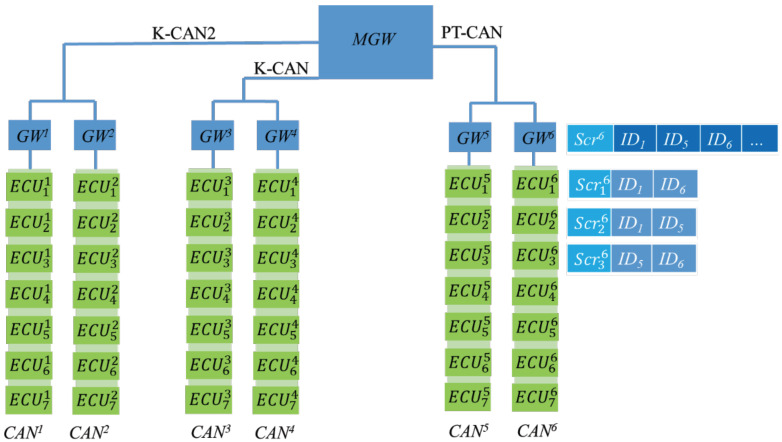
Modified in-vehicle system.

We segmented high-speed and low-speed CAN bus networks into subnets on the basis of real-time demands and placed ECUs requiring high-speed sessions on the same CAN. Each CAN is equipped with a gateway ($GW_{1}$) responsible for distributing keys and transmitting to external CAN bus networks. If the transmitter and target ECU are both on the same CAN bus network, the message is transmitted directly. If the transmitter and target ECU are on different CAN bus networks, the message is broadcast through the area network. Because CAN bus message IDs are used to indicate the message type, the target ECU receives messages on the basis of the required ID. In the proposed framework, each GW has a table listing all the required IDs in its area. The gateway $GW_{1}$ broadcasts the message to the gateway group, and gateway $GW_{2}$ looks for IDs in its own table and then broadcasts the message with that ID to its own area. The system framework is presented in Figure 9.

In this figure, $GW_{6}$ has a subscription table storing $ID_{1}$, $ID_{5}$, and $ID_{6}$. When $GW_{6}$ receives messages with these IDs in the GW group, it broadcasts these messages in its own area.

### 4.2. Notations

Table 3 presents the symbols used in the protocol.

### 4.3. System Initialization

**Figure 10:**
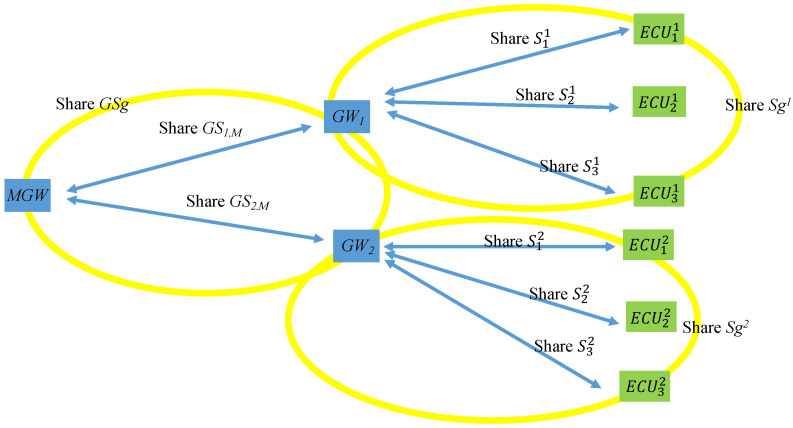
Factory setting keys.

In a vehicle’s factory settings, the key shared by the MGW and each $GW^{i}$ is $GS_{i,M}$. Each $GW^{i}$ shares a group key with every $ECU_{j}^{i}$ in its CAN, which is denoted as $S_{g}^{i}$, and shares individual keys with each ECU, which are $S_{1}^{i}$, $S_{2}^{i}$, $S_{3}^{i}$, and so on. Figure 10 illustrates the keys shared among the MGW, the GWs, and the ECUs.

### 4.4. System Reset

When the engine is first activated, the MGW broadcasts a “time synchronization” frame to all the GWs, and the GW time counters are reset to zero. This message is sent using a specified ID, which is 00000000000 (time sync ID), and the original CRC. The GW then broadcasts the same frame to its CAN, causing all the ECUs to reset their time counters to zero upon receiving the broadcast. This frame is only sent at this time. None of the ECUs and GWs will accept this frame again after receiving this frame for the first time until the engine is restarted. This prevents attacks from resetting the time counters.

### 4.5. First Distribution of Keys

**Figure 11:**
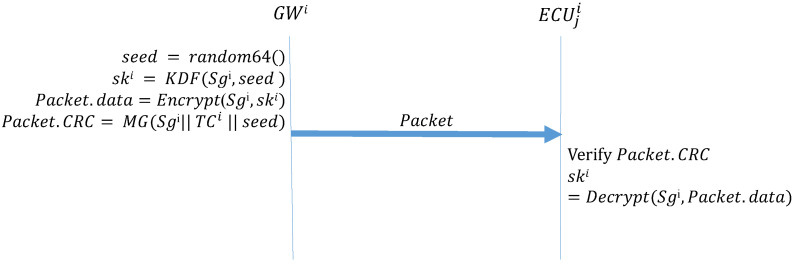
Initial group key configuration by GW and ECUs.

Algorithm 1 (*GW_Startup*) describes the function that the GW side performs during the initial distribution of keys after system activation. First, the GW side generates a 64-bit random number seed; next, it uses the preset group key and the seed in the KDF to create a group key. The KDF directly performs a hash operation on the received seed to obtain the group key for the next session. The preset group key is then used to encrypt the next group key to be used, which is placed in the data field of the frame being transmitted; next, MG is used to create the MAC. The MG function, which was introduced in Section 3, uses data in the ID, DLC, and data fields, the preset group key shared by all the ECUs, and the current time counter to create a MAC, which is stored in the CRC field of the frame. Finally, the frame is broadcast to every CAN. Algorithm 2 (*ECU_Startup*) describes the function to be performed by the ECUs during the initial distribution of keys. First, an ECU creates a MAC by using the data it receives, its current time counter, and the preset group key. If this MAC is consistent with the MAC in the CRC field of the received frame, the ECU decrypts the data field of the frame to obtain the session key. Figure 11 presents the process for configuring the first group key.
**Algorithm 1:** GW_Startup-Initial distribution of keys: GW broadcast of the group key.**Input**: ${S_{g}}^{i}$, $TC^{i}$ **Output**: Packet, $sk^{i}$
seed = random64() ;
$sk^{i}$ = $KDF({S_{g}}^{i},seed)$;
Packet.data = $Encrypt({S_{g}}^{i},sk^{i})$ ;
Packet.CRC = $MG({S_{g}}^{i}∥TC^{i}∥seed)$;


**Algorithm 2:** ECU_Startup-Initial distribution of keys: ECUs receive the group key.**Input**: ${S_{g}}^{i}$, $TC^{i}$, Packet **Output**: $sk^{i}$ $MAC=MG({S_{g}}^{i}∥TC^{i}∥Packet.data)$; 
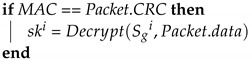


### 4.6. Message Transmission

**Figure 12:**
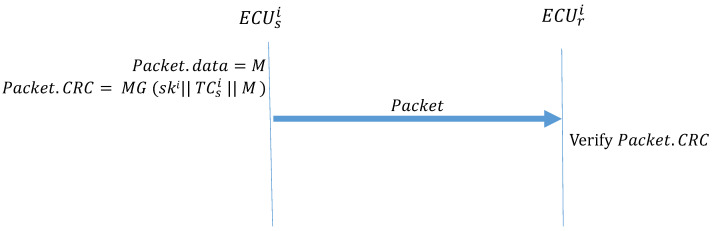
ECUs transmitting frames within the CAN.

**Figure 13:**
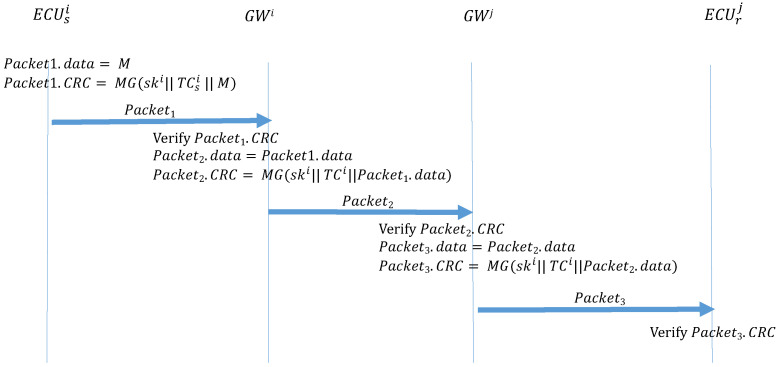
ECU frame transmission outside the CAN.

Algorithm 3 (Message_Transmission) describes the function performed when two ECUs in the same subnet $CAN_{i}$ transmit data. The transmitter uses MG to create a MAC and places it in the CRC field of the frame. When $ECU_{r}$ receives the frame, it first calculates the MAC and confirms whether it is consistent with the MAC in the CRC field of the frame. If the MACs are not the same, the message is discarded. If the MACs are the same, the authentication is successful, and the frame is accepted. Figure 12 illustrates how the ECUs transmit frames in the CAN.
**Algorithm 3:** Message_Transmission**Input**: M, $TC_{s}^{i}$, $sk^{i}$ **Output**: Packet
Packet.data = M ;
Packet.CRC = $MG({sk}^{i}∥TC_{s}^{i}∥M)$;


If the transmitter and the receiver are in different CANs, two additional authentication steps are required between the GWs. Algorithm 4 (ECU_Forwarding) describes the function that is performed by a transmitter to send out the frame. The transmitter uses MG to create a MAC and places it in the CRC field of the frame, which is then broadcast to the subnet where the transmitter is located. Algorithm 5 ($GW_{s}\_Forwarding$) describes the function performed by the GW of the transmitter subnet to receive this frame and sends it to the GW group. When GW receives this frame, it first calculates the MAC and confirms whether it is consistent with the CRC field of the frame. If the frame is authenticated, the GW uses MG to create a MAC and places it in the CRC field of the frame, then broadcasts the frame to the GW group. Algorithm 6 ($GW_{r}\_Forwarding$) is the function performed by the GW of the receiver subnet when it discovers the ID of the frame in its subscription table and broadcasts the frame to the receiver subnet. When the receiver GW receives the frame, it also calculates the MAC and confirms whether it is consistent with the CRC field of the frame. If the MACs are authenticated, the GW uses the MG to create a MAC, which is placed in the CRC field of the frame, then broadcasts the frame to the receiver subnet. Finally, after the receiver receives the frame in the subnet, the receiver calculates the MAC and compares it against the CRC field. If the MACs are not consistent, the frame is discarded. If the MACs are consistent, the authentication is successful, and the frame is accepted. Figure 13 illustrates the process.
**Algorithm 4:** ECU_Forwarding: ECU transmissions between subnets.**Input**: M, $TC_{s}^{i}$, $sk^{i}$ **Output**: $Packet_{1}$ $Packet_{1}.data=M$ ;
$Packet_{1}.CRC=MG({sk}^{i}∥TC_{s}^{i}∥M)$;


**Algorithm 5:**$GW_{s}\_Forwarding$: GW transmissions between subnets.**Input**: $TC^{i}$, $sk^{i}$, Gsk, $Packet_{1}$ **Output**: $Packet_{2}$
MAC = $MG({sk}^{i}∥TC^{i}∥Packet_{1}.data)$ ;

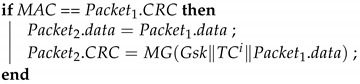



**Algorithm 6:**$GW_{r}\_Forwarding$: GW transmissions between subnets.**Input**: $TC^{j}$, Gsk, $sk^{i}$, $Packet_{2}$ **Output**: Packet3
MAC = $MG(Gsk∥TC^{j}∥Packet_{2}.data)$ ;

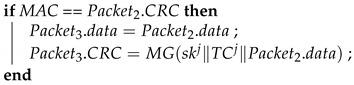



### 4.7. Key Updates

**Figure 14:**
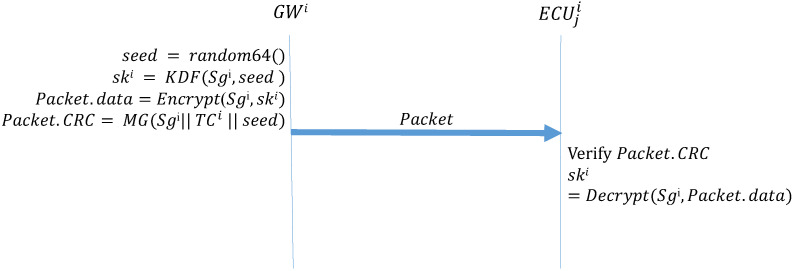
Updating session keys in each interval.

Occasionally, the session group keys must be replaced. At this time, GW generates a new seed and then uses the previous session group key to create a MAC, which is then broadcast to the ECUs on the subnet. Algorithm 7 (*GW_KeyUpdate*) describes the function that the GW side performs when updating keys. The GW side first generates a 64-bit random number seed and then uses KDF to create a group key. The GW then uses the preset group key to encrypt the pending group key, which is placed in the data field of the frame to be transmitted. Next, MG is used to create a MAC, which is placed in the CRC field of the frame. Finally, this frame is broadcast to every ECU in the CAN. Algorithm 8 (*ECU_KeyUpdate_Reponse*) describes the function that the ECUs perform during the initial distribution of keys. First, a MAC is created. If this MAC is consistent with the CRC field of the received frame, the ECU decrypts the data field of the frame to obtain the session group key. Figure 14 depicts the process of updating the session key.
**Algorithm 7:**GW_KeyUpdate: Key updates: GWs.**Input**: ${S_{g}}^{i}$, ${TC}^{i}$ **Output**: Packet, ${sk}^{i}$
seed = random64() ;
${sk}^{i}$ = KDF(${S_{g}}^{i}$, seed) ;
Packet.data = Encrypt(${S_{g}}^{i}$, ${sk}^{i}$) ;
Packet.CRC = $MG({S_{g}}^{i}∥TC^{i}∥seed)$ ;


**Algorithm 8:**ECU_KeyUpdate_Response: Key updates: ECU.**Input**: ${S_{g}}^{i}$, ${TC}_{j}^{i}$, Packet **Output**: ${sk}^{i}$
MAC = $MG({S_{g}}^{i}∥TC_{j}^{i}∥Packet.data)$ ;

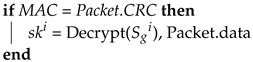



### 4.8. Adding a Device

**Figure 15:**
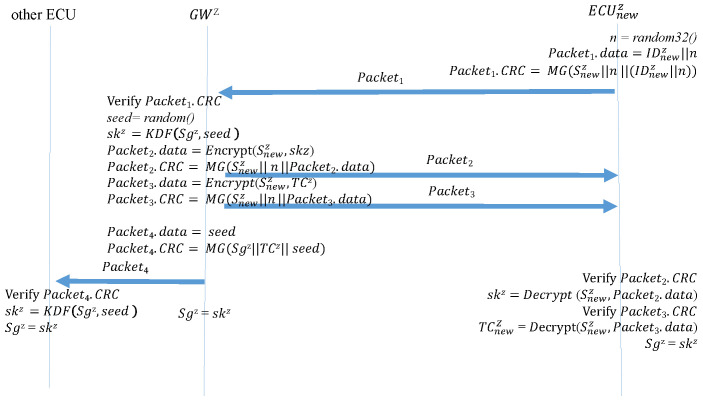
Adding a device to the CAN bus.

After a device is added, the new device sends a request to the GW to join the subnet. Algorithm 9 (Join_Request) is the function performed by the new device when sending this request. The new device generates a 32-bit random number, which is written in the data field along with the device ID. The random number is then used to replace the original time counter. A MAC is generated using the preset group key and then sent to the GW. The random number prevents the frame from being replayed before the new device can reproduce the time counter. Algorithm 10 (Join_Response) is the function that the GW performs after receiving a join request from the new device. After receiving and authenticating the MAC, the GW generates a new seed and uses the seed to create a new group key. It then encrypts the group key and a time counter in the data fields of two separate frames while using the random number from the new device in the original time counter location to create two MACs. These are sent separately to the new device. Next, the encrypted new group key and MACs are broadcast to the other ECUs as a key update. Finally, the new session group key overwrites the preexisting group key. Therefore, the new members can participate in the key update. Algorithm 11 (Join_TakeKey) is the function performed by the new device after it receives the two responding frames from the GW. The new device first calculates the new MACs and determines whether they are consistent with the CRC fields of the two frames. If so, the new device decrypts the data fields of the two frames to obtain the group key and the time counter, enabling the new device the join sessions. Finally, the new device overwrites the preset group key with the session group key. Algorithm 12 (Join_UpdateKey) is the function performed by the other ECUs in the subnet when receiving a new group key. After receiving the frame, the ECUs calculate the MAC. After the MAC is confirmed to match the frame CRC field, the ECUs decrypt the data field and obtain the new group key. Finally, the preexisting group key is overwritten with the new group key. Figure 15 depicts the process of a device joining the CAN.
**Algorithm 9:**Join_Request: the new device sends a join request.**Input**: ${ID}_{new}^{i}$, $S_{new}^{i}$ **Output**: $Packet_{1}$
n = random32(); ;
$Packet_{1}.data={ID}_{new}^{i}∥n$ ;
$Packet_{1}.CRC=MG(S_{new}^{i}∥n∥Packet_{1}.data)$ ;


**Algorithm 10:**Join_Response: the GW responds to the join request.**Input**: $Packet_{1}$, $S_{new}^{i}$, ${TC}^{i}$, ${S_{g}}^{i}$ **Output**: $Packet_{2}$, $Packet_{3}$, $Packet_{4}$
MAC = $MG(S_{new}^{i}∥n∥Packet_{1}.data)$ ;

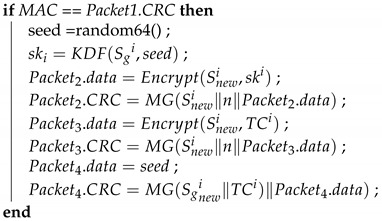



**Algorithm 11:**Join_TakeKey: The new device obtains the group key and time counter.**Input**: $Packet_{2}$, $Packet_{3}$, $S_{new}^{i}$, n **Output**: $sk^{i}$, ${TC}_{new}^{i}$, $S_{g}$
MAC1 = $MG(S_{new}^{i}∥n∥Packet_{2}.data)$ ;

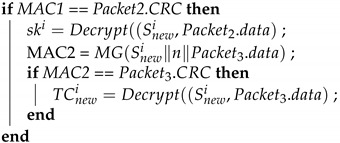



**Algorithm 12:**Join_UpdateKey: The ECUs update their group key.**Input**: $Packet_{4}$,${S_{g}}^{i}$, ${TC}_{j}^{i}$ **Output**: $sk^{i}$, , $S_{g}$
MAC = $MG({S_{g}}^{i}∥{TC}_{j}^{i}∥Packet_{4}.data)$ ;

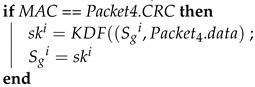



### 4.9. Removing a Device

**Figure 16:**
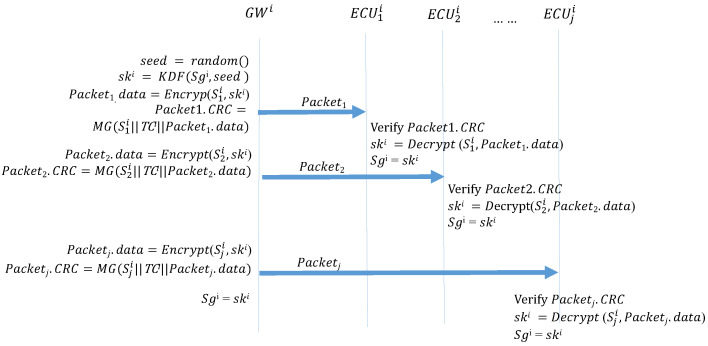
Removing a device from the CAN bus.

When removing a device, the GW side sends an order to all the ECUs in the subnet to update the group key. The Algorithm 13 (*Leave_KeyChange*) is the function that the GW performs when removing a device. The GW first generates a new seed and uses KDF to generate a new group key. At this time, the previously distributed group key is not used; instead, the previously distributed individual keys are used to encrypt the new group key and create MACs using MG. These are sent separately to each ECU on the subnet. Finally, the preexisting group key is overwritten with the new group key. Algorithm 14 (*Leave_Response*) describes the function performed by each ECU after receiving this frame. First, the ECU calculates the MAC; if the MAC matches the CRC field of the frame, the authentication is successful, and the ECU decrypts the new group key. Finally, the preexisting group key is overwritten with the new group key. The removed device is then unable to participate in the key updates. Figure 16 depicts the device removal process.
**Algorithm 13:**Leave_KeyChange: The GW sends out the group key individually.**Input**: ${S_{g}}^{i}$, $S_{j}^{i}$, ${TC}^{i}$ **Output**: Packet , $S_{g}$
seed = random64(); ;
$sk^{i}=KDF\left({S_{g}}^{i},seed\right)$ ;
Packet.data = $Encrypt(S_{j}^{i},sk^{i})$ ;
Packet.CRC = $MG(S_{j}^{i}∥{TC}^{i}∥Packet.data)$ ;
${S_{g}}^{i}={sk}^{i}$ ;


**Algorithm 14:**Leave_Response: ECUs receive the group key individually when a device is removed.**Input**: Packet,${S_{g}}^{z}$, ${TC}_{j}^{z}$ **Output**: $sk^{z}$, ${S_{g}}^{z}$
MAC = $MG(S_{j}^{i}∥{TC}^{i}∥Packet.data)$ ;

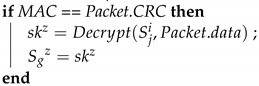



## 5. Security Analysis

Of the numerous types of CAN bus attacks, our discussion focuses on spoofing and replay attacks. In addition, we discuss desynchronization between transmitters and receivers.

(A) Spoofing attacks: in a typical environment, the frame itself only has CRC protection. Attackers spoof frames on the CAN by using the incorrect data field and deriving the CRC; these spoofed frames can be successfully accepted by an ECU. By adding a MAC to verify the source, attackers must spoof both the frame and the MAC for their spoofed frame to be accepted. Furthermore, attacks must have both the current group key and the current counter to generate the correct MAC. The process for sending keys only involves sending the seed to make the key, and attackers cannot obtain the formula for generating keys; therefore, attacks cannot gain access to the current key. Our MAC only has 15 bits, and attackers attempting to forcibly decrypt the MAC must run through 215 possibilities. Even if the attacker attempts every possibility, they are limited by the slow speed of the CAN bus itself; that is, before running through all the possibilities, the next frame will have reached the target end. This is how our method effectively defends against spoofing attacks.
(B) Replay attacks: Because the frame itself does not have an authentication mechanism, ECUs accept any recorded frame. In our protocol, each message being transmitted creates a MAC on the basis of the current time counter, and different time counters result in different MACs. As such, if an attacker records a previous frame and transmits it, it is not accepted by the ECU. The time counter is involved in both the initial and subsequent transmission of keys to increase the freshness of the MACs. If an attack records a previous frame, because of the different time counter, the prerecorded frame is not successfully verified. In this protocol, the only frame without a time counter or MAC is the reset frame sent out in the beginning; however, this frame is received only once by all the ECUs at the reset and is not accepted or executed again. Consequently, replaying the reset frame does not have any effects. Replay attacks are possible between custom time intervals Δt, and therefore, Δt must be designed in consideration of the CAN speed to impede attacks from launching replay attacks between the time intervals.
(C) Desynchronization: we were concerned about the problem of desynchronization between ECUs in other protocols, whether through desynchronized counters or desynchronized keys. Desynchronized counters directly lead to authentication failures. In the CAN bus, receivers often miss frames, especially in situations with an arbitration mechanism. Essentially, multiple methods involve resetting counters while synchronizing keys to ensure that the time counters synchronize after each interval. However, missed frames still occur, and one missed frame can cause the counter to desynchronize, leading to continued verification failures. Therefore, we chose to use time counters, enabling the counter to always be synchronized. Even if each ECU had a slightly different reset time, resulting in some differences in the time counters, when the receivers fail to authenticate a frame by using its current time counter to calculate the MAC, the ECU attempts to add to or subtract from the time counter to authenticate packets lost because of discrepancies in the time counters. This method does not lead to desynchronized time counters because of missed packets. The PreAuthCode method by which individual keys update their own keys was also considered, but it cannot fully ensure that every counter is perfectly synchronized—that is, if one counter is different from the others, it can no longer synchronize. Therefore, we decided that synchronization would be uniformly performed by the GWs sending out a new seed at fixed times. These two mechanisms in operation ensure that authentications are performed normally even if the counters are slightly desynchronized and that the keys are always synchronized.

## 6. Experimental Result

**Figure 17:**
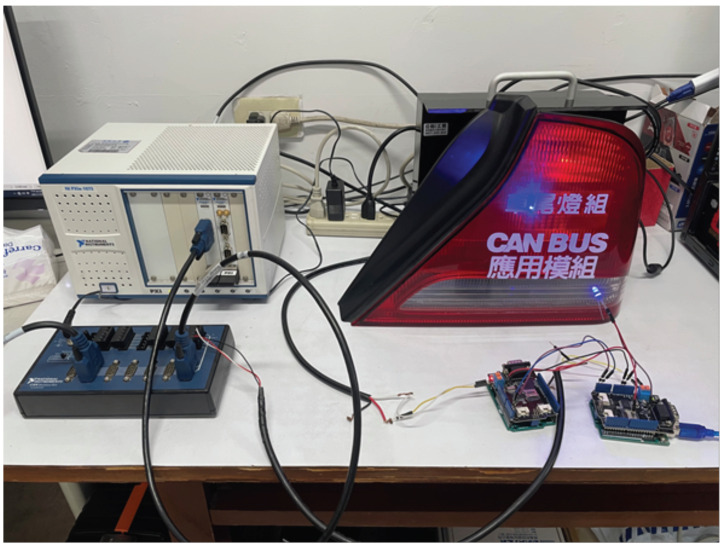
The experimental framework.

Our emulation environment is mainly divided into three parts—the core CAN-bus network, the peripheral equipment and the add-in security module for each ECU. The overall structure is shown in Figure 17.

We use the CAN shield 2.0 and connects it to the OBD II interface in the car to capture the CAN bus packets sent by a NISSAN TEANA 2.0 (2010 model) as the background traffic.

We use National Instrument (NI)’s PXIe-1073 as the core network. A computer acts as the main gateway is connected to PXIe-1073 through the PCIe 8361 MXI Express interface; in addition, through the PXI-8512 PXI CAN interface card module and a CAN breakout box to simulate our CAN-bus environment. The breakout box is used to emulate the connection of several independent CAN-buses. Therefore, several peripheral devices on the real car are used to build an emulated environment. These peripheral devices include taillights, headlights, antennas and doors. Each peripheral device listens to a specific CAN’s ID address.

**Figure 18:**
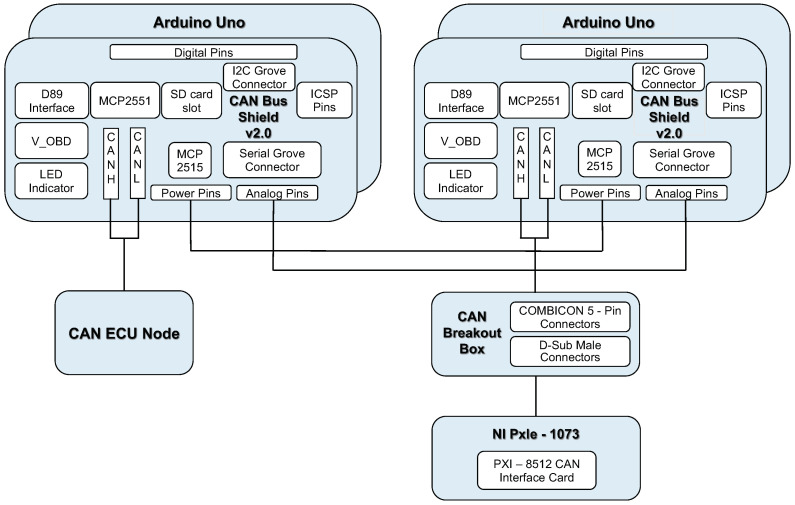
The add-on security module.

Next, we use two Arduino UNOs equipped with CAN BUS Shield 2.0 expansion board as the add-on security module between the can-bus network and peripheral devices’ ECU units, as shown in Figure 18. We implemented the proposed communication protocol on the Arduino UNO boards, so that the boards can send and receive messages from CAN-bus. When the packet received by the first Arduino UNO contains the correct message authentication code in the CRC field, the packet will be forwarded to the second Arduino UNO board.

When the second Arduino Uno board receives the packet, it calculates the CRC field based on the original CAN protocol, and uses it to replace the MAC field in the received packet. After that, the packet will be forward to the physical devices via the CAN BUS Shield 2.0 expansion board.

If the message authentication code of the message is wrong, the packet will be directly discarded by the first Arduino Uno board. In this case, no packets will be sent to the second Arduino Uno board. Therefore, for the actual peripheral devices of the car, the wrong packet will not be received.

**Figure 19:**
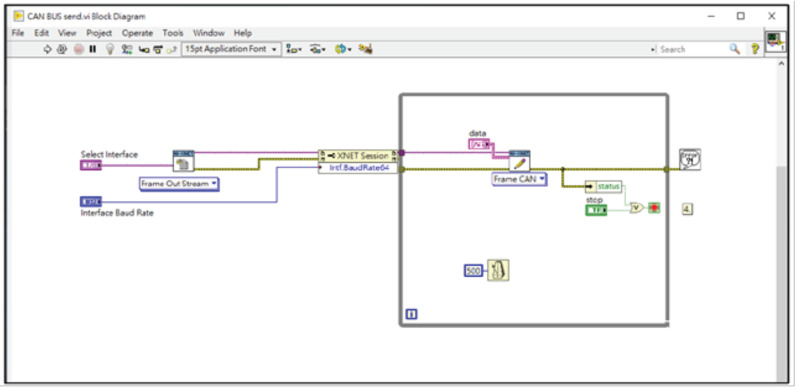
The LabVIEW codes executed on the NI PXIe-1071.

In order to be able to simulate the operation of the entire system, we wrote LabVIEW programs on the NI PXIe-1073 machine to simulate the operation of the main gateway and the injection of packets, as shown in Figure 19. The injection packets are modified from the background traffic by using the proposed algorithms to calculate the new MAC field of the packets, and then pass the modified packets into our experimental network through the PXIe-1073 and the PCIe 8361 MXI Express interface.

**Figure 20:**
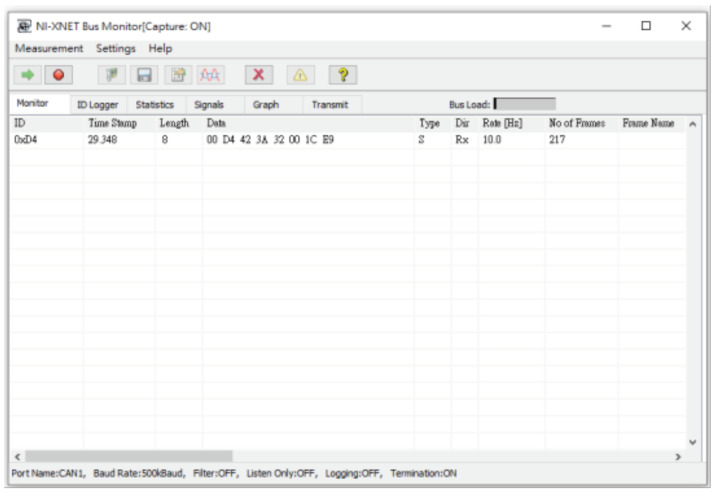
The NI-XNET Bus monitor.

In addition, we also added attack packets and passed them into our experimental module to simulate denial of service attacks. We send normal packets and attacks packets into the network without orders, and uses the build-in monitoring software NI-XNET BUS Monitor to monitor all packets on the CAN BUS, as shown in Figure 20.

In our experiment, we insert malicious packets in the CAN bus up to 500 kbps. Our system can detect these erroneous packets and discard them so that it will not affect the operation of the CAN-bus.

In addition, when an attack occurs on a CAN bus, the Gateway ECU will detect these problematic packets and discard them, these packets will not be forwarded to other CAN-bus networks. Therefore, this attack will be limited to the source CAN-bus, and the performance of the overall CAN-bus system will not be affected.

## 7. Implementation in the CAN-FD Environment

**Figure 21:**
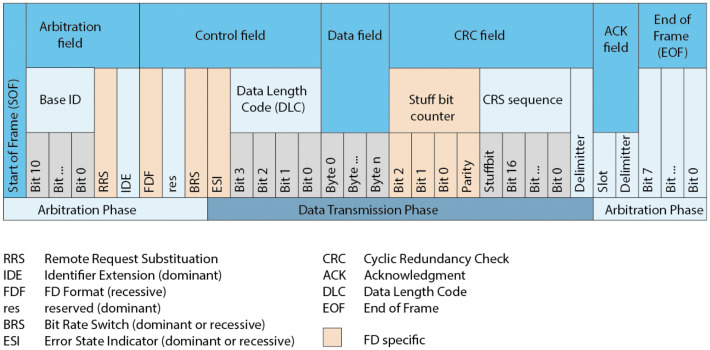
Th structure of the CAN-FD frame [49].

In the above section, we implement the system in the traditional CAN frame with the modification of CRC field. However, when the incompatible node uses the original CRC algorithm to verify the packet, an error will occur and the packet may be dropped. In this section, we show how to apply our proposed algorithm to the CAN-FD architecture. In the CAN-FD architecture, the data length of each packet is recorded in the *Data length Code* (DLC) of the control field, which is up to 64 bytes, as shown in Figure 21.

When our method is implemented in CAN-FD, we assume that the maximum length of the data field will not exceed 62, and two extra bytes contain the MAC will be inserted into the end of the data field. We also change the *res* bit in the control field to 1 (this means that we have added MAC data in the data field) and increase the value of *DLC* by 2.

Algorithm 15 describes the function performed when two ECUs in the same subnet $CAN_{i}$ transmit data, which is the modified version of Algorithm 3.
**Algorithm 15:** CAN-FD_Message_Transmission.**Input**: M, $TC_{s}^{i}$, $sk^{i}$ **Output**: Packet
Packet.data = M ;

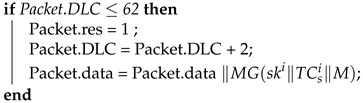



The rest of our protocol is the same as described in the above section.

## 8. Conclusions

In this paper, a lightweight authentication protocol for CAN bus was proposed. Due to the limitation that the data field of a CAN frame is limited to 8 bytes, we use a novel message authenticate code to replace the original CRC field in the frames of the CAN protocol.

To synchronize the time of each ECU, we propose an algorithm that can self-correct the time difference. By using our time counter mechanism, each ECU will self-correct the time difference with other ECUs. Even if the discrepancy between the ECUs is greater than 1 s per hour, authentications can be successfully completed using our protocol. Our proposed mechanism can perform synchronizations and authentications while using fewer resources. We also used group keys to reduce the amount of resources required to send keys. Only one broadcast is necessary to distribute or update keys among the subnet ECUs. Partitioning the CAN system can further reduce the burden on the main gateway, and it is more stable than methods managed by only one main gateway.

Comparing to other protocols, which use the extended identifier field and the data field in the CAN frames, our proposed protocol does not require additional packets and data fields to authenticate the message.

In the experimental results, we use packets from a real car to simulate the operation of the entire framework. We have also proved that the proposed method can be used in CAN networks. The future development of the CAN bus should be CAN-flexible data rate (FD)–centric [43]. Our method can also be applied in CAN-FD environments, even in systems that run CAN bus and CAN-FD in parallel. The proposed method is anticipated to be successfully implemented on CAN-FD.

## Figures and Tables

**Table 1:** Types of error frames.

Error Type	Description
bit error	The transmitted data is inconsistent with what is presented in the bus; therefore, an error frame is sent out.
stuff error	When six consecutive identical bits are detected, an error frame is sent out.
CRC error	An error frame is sent out in response to a CRC error.
form error	If the delimiter bit is 0, an error frame is sent out, starting with the next bit.
acknowledgment error	The transmitter sends out an error frame if a response is not received after the ACK field.

**Table 2:** Methods for gaining complete control of a vehicle with CAN bus data routing and ECU updates [13].

Channel of Infiltration	Method of Implementation	Scope
OBD-II interface	Connects to the attack device directly through the OBD-II interface	Small
CD player	Installs malware through updates	Small
PassThru automotive reprogramming device	Connects to a reprogramming device through WiFi and installs malware	Large
Bluetooth	Installs malware through overflow attacks	Large
Bluetooth	Eavesdrops on the message authentication code (MAC) address and forcibly decrypts the PIN	Small
Telephone network	Uses a laptop or smart phone to access the automobile and install malware through overflow attacks	Large

**Table 3:** Notations.

Symbol	Description
$CAN^{i}$	The *i*th group under CAN bus.
$GW^{i}$	The gateway that manages the $CAN^{i}$ bus.
MGW	The main gateway that manages all gateways.
$ECU_{j}^{i}$	The *j*th ECU in the $CAN^{i}$ bus.
$S_{j}^{i}$	The pre-shared key between $ECU_{j}^{i}$ and $GW^{i}$.
M(message)	The sent frame.
${S_{g}}^{i}$	The pre-shared keys for all ECUs and the $GW^{i}$ in the $CAN^{i}$ bus.
$GS_{i,M}$	The key shared by MGW and $GW^{i}$.
$sk^{i}$	The session key used by the ECUs in $CAN^{i}$ bus.
Gsk	The session key used in the GW group.
